# Cultivating cultural capitals in introductory algebra-based physics through reflective journaling

**DOI:** 10.1103/physrevphyseducres.18.020139

**Published:** 2022-11-29

**Authors:** Khanh Tran, Ana Maria Barrera, Kim Coble, Mireya Arreguin, Marissa Harris, Alex Macha-Lopez, Michaela Perez, Alegra Eroy-Reveles

**Affiliations:** 1 Purdue University, West Lafayette, Indiana 47905, USA; 2 San Francisco State University, San Francisco, California 94132, USA; 3 UC Santa Cruz, Santa Cruz, California 95064, USA

## Abstract

At a large, diverse, hispanic-serving, master’s-granting university, the Alma Project was created to support the rich connections of life experiences of science, technology, engineering, and mathematics (STEM) students that come from racially diverse backgrounds through reflective journaling. Utilizing frameworks in ethnic studies and social psychology, the Alma Project aims to make learning STEM inclusive by affirming the intersectional identities and cultural wealth that students bring into STEM classrooms. Approximately once per month students who participate in the Alma Project spend 5–10 min at the beginning of class responding to questions designed to affirm their values and purpose for studying STEM in college. Students then spend time in class sharing their responses with their peers, to the extent that they feel comfortable, including common struggles and successes in navigating through college and STEM spaces. For this study, we analyze 180 reflective journaling essays of students enrolled in General Physics I, an algebra-based introductory physics course primarily for life science majors. Students were enrolled in a required lab, a self-selected community-based learning program (Supplemental Instruction), or in a small number of instances, both. Using the community cultural wealth framework to anchor our analysis, we identified 11 cultural capitals that students often expressed within these physics spaces. Students in both populations frequently expressed aspirational, attainment, and navigational capital, while expressions of other cultural capitals, such as social capital, differ in the two populations. Our findings suggest that students bring rich and diverse perspectives into physics classrooms when asked to reflect about their lived experiences. Moreover, our study provides evidence that reflective journaling can be used as an asset-based teaching tool. By using reflective journaling in physics spaces, recognizing students’ assets has the potential for physics educators to leverage students’ lived experiences, goals, and values to make physics learning more meaningful and engaging.

## I. INTRODUCTION

### A. Uplifting the experiences of marginalized students in STEM

Students marginalized along one or more dimensions of race or ethnicity (e.g., African American, Native American, Hispanic, or Latino/a, Southeast Asian, and Pacific Islander), gender, sexual orientation, (dis)ability status, socioeconomic status, etc., often face hardships as they navigate through the United States educational system. In addition to access issues, many marginalized STEM students, including underrepresented minorities (URM), experience harassment, microaggressions, stereotype threat, and lack of support from faculty during their time in college [1–3]. These processes exacerbate underrepresentation (e.g., for URM students compared to their White counterparts) as marginalized students climb the higher education ladder [4–6] and many are eventually pushed out of the “STEM pipeline” [7]. This is particularly true for students who identify as Black and Latino/a and for students of multiply marginalized intersectional identities [2,8–10].

Research focusing on improving diverse students’ experiences has been a growing priority in the physics and astronomy education community, including naming and acknowledging the issues, affirming students’ multiple identities, and engaging in collective actions to remove barriers that can support access and persistence [11]. Based on a large body of research, the physics and astronomy community has put forth a number of recent reports and recommendations to address inequities and improve inclusion (e.g., Refs. [12–15]), including adoption of teaching practices that support marginalized students. Furthermore, there is a need to study diverse student populations and course contexts, as the current physics education research (PER) literature has been disproportionately centered at primarily white institutions and in the calculus-based physics course [16].

Scholars in PER have aimed to understand the experiences of marginalized, historically underrepresented people in physics spaces in a number of ways (see, e.g., special journal collections [17–19], resource letters [20], and examinations of universal design [21]). Studies have been conducted at all levels (K-12, undergraduate, graduate, professional) and have looked at the relationships between various combinations of marginalization and performance, persistence, attitudes, identity, self-efficacy, sense of belonging, learning environment, and other constructs. These investigations illuminate the complex challenges people often endure when navigating through physics and other academic spaces. For example, Cwik and Singh [22] explored the role of gender and students’ insight on their learning environment in algebra-based courses for bioscience majors and found that perceived recognition, peer interactions, and sense of belonging predict self-efficacy, interest, identity, and grades. Therefore, they recommend that instructors “create a learning environment that emphasizes recognizing their students, allowing for positive peer interactions, and providing a space where all students can feel like they belong in physics.” Physics education scholars have also examined changes in students’ attitudes about physics quantitatively as a function of race or ethnicity, gender, and intersections thereof in various introductory course contexts [23,24]. Using their critical physics identity framework, Hyater-Adams *et al*. [25] explored factors that influenced the ways Black people (of various genders or sexual orientations) identify with physics. They argue that efforts to support students go beyond material resources to include ideational resources, i.e., ideas and ideologies about themselves and the field, which can impact students’ participation in physics. They recommend “intentional effort[s] to break down harmful ideologies” in physics culture, affirming students’ views of themselves, and cultivating positive recognition. Studies shedding light on intersectional experiences emphasize attention to complexity, moving away from deficit framings, and highlighting marginalized voices and lived experiences, including success and joy, while acknowledging the institutional barriers that exist in physics, astronomy, and other STEM spaces [26–29].

Defining inclusive practices and identifying specific ways in which physics educators and researchers can support marginalized students is an important topic of study. For example, Mitchell-Polka *et al*. [30] developed a framework for empowerment in physics education as a tool to engage students and improve access to STEM education for all groups, arguing that empowering students through various dimensions (i.e., social interaction, academic relationships, etc.) can create new pathways that make physics more accessible. Instructors also play a critical role in maintaining students’ motivation and making STEM classrooms inclusive [31] and increasing students’ sense of belonging [32]. By engaging in dialogue and personal reflections, faculty can become more aware of the inequities that continue to exist in STEM spaces. One such way scholars have envisioned to improve the experiences of URM students learning physics includes adopting teaching practices grounded in a resource perspective, based on strengths of URM students not typically found in majority populations or through traditional assessments [33]. By listening to students’ lived experiences and noticing students’ strengths, physics educators can then make learning environments more equitable by changing classroom dynamics, critiquing institutional practices, and validating students’ identities and representation in STEM [34]. Teaching about equity as part of the physics curriculum [35,36] and engaging in critical work can also be a powerful tool to address access and inclusion for URM students.

While previous studies offer important insights in how marginalized students navigate through physics and ways to support them, there is still more work to be done in identifying specific assets and skills gained through overcoming adversities that are important to leverage in physics spaces. Prior studies provide strategies to make teaching physics more equitable and accessible, but many interventions have primarily grown out of the perspectives of researchers and instructors, or from a deficit approach. In turn, the lived experiences of URM students often go unacknowledged and underincorporated in the classroom. For example, a study of instructors of introductory physics for life sciences (IPLS) classes [37] found that faculty often relied on other faculty or assumptions about what students would need to know for their careers to make curricular choices, rather than directly engaging students. They point out the opportunity this presents for students’ voices to shape the future directions of such courses.

The Alma Project was initiated by students, for students, and centers the perspectives of students. When instructors learn about the stories of their students, they can become more equitable by being culturally responsive to their needs [38]. If instructors affirm students’ identities and acknowledge their frames of reference and assets, students cannot only gain access and achievement as part of an existing status quo, but they can grow their power and become critical citizens capable of “changing the game” [39]. Through the implementation component of the Alma project in the classroom, we aim to affirm our students’ diverse identities and experiences; through the research component, we aim to elevate our students’ voices to help instructors acknowledge and leverage a wider range of students’ assets than has traditionally been recognized in physics spaces.

### B. Critical race theory

Drawing on critical race theory (CRT), we recognize the interplay of racism, power, and how systemic barriers continue to hinder the access of URM students, by creating conditions where they endure more educational hardships and challenges than their White counterparts [40,41]. CRT frames an understanding of race as a social construction and urges us to center race, racism, and power in our examinations, given that Students of Color have been historically and presently excluded in various spaces [40,41], including higher education and STEM. More specifically in education, Yosso notes that, “CRT in education refutes dominant ideology and White privilege while validating and centering the experiences of People of Color” [42]. CRT is grounded on five critical tenets [40] outlined in Table I. Together, these tenets shape our perspectives on the social constructs of race, racism, and the interplay with other forms of marginalization in various institutions and spaces. Our work heavily draws on the first and fourth tenets given that we are focusing on marginalized students’ voices through reflections and emphasize the importance of lived experiences as important assets in navigating racialized spaces.

### C. Community cultural wealth framework

From a CRT perspective, students respond to marginalization with the motivation to succeed by persisting and overcoming dominant narratives while uplifting their families and communities. The challenges that marginalized students endure due to an inequitable and unjust system in turn cultivate richness in experiential knowledge gained from persisting through systems of oppression, resulting in assets that students can utilize in navigating through their educational journey [43]. To further this critical perspective in the context of the introductory physics classroom, the Alma Project centers on the voices of STEM students by utilizing reflective journaling as a way for them to narrate their lived experiences and values. We draw on Yosso’s community cultural wealth (CCW) framework [42] to situate how we view students’ experiences and the experiential knowledge they bring into the classroom.

In alignment with the importance and richness of experiential knowledge, CCW is an asset-based framework that identifies the strengths and cultural repertoires that URM students bring to the classroom. Cultural capitals are nurtured in students’ communities and contribute to their persistence and academic success [42]. Yosso identified six cultural capitals—aspirational, linguistic, familial, social, navigational, and resistant—that are often nurtured within URM students by their communities. The conceptualization of CCW reframes cultural knowledge, skills, and abilities that URM students possess as an important part of learning physics rather than abilities that are irrelevant to their educational journey. Because of the large number of Latino/a/x students enrolled at our university, we also employed Kanagala and colleagues’ contribution to the CCW framework [44] that includes perseverant and spiritual capital.

Using CCW as a framework in STEM classrooms makes learning relevant to students’ experiences and can be a motivational factor for persistence in STEM programs [45–48]. A sense of belonging can also be cultivated in higher education STEM classrooms when students’ community cultural wealth is incorporated into the classroom [49]. The use of reflective journaling in STEM spaces can also allow students to identify and recognize their own forms of CCW. There is a huge opportunity in physics for educational practices and research investigations that utilize CCW. In their study of successful black women physicists, Rosa and Mensah emphasize the importance of counter storytelling [29], a premise of CRT highlighting the experiential knowledge and voices of people who are not usually heard, through narrative forms as a way of disrupting dominant narratives (e.g., only White men can do physics) and entrenched power structures. They point out that “educators can make use of storytelling to unveil and validate the experiences of students of color in science” and “physics educator researchers can use this tradition as a methodological tool to expand the community’s knowledge on the experiences of people of color in STEM fields.” Understanding what kinds of assets students are utilizing can empower them to cultivate a space in physics where content knowledge is met with lived experiences. Journaling in physics spaces can serve as a tool to validate students’ experiences and therefore, we use reflective journaling in physics classrooms to address this opportunity.

### D. Reflective journaling

In physics education, reflective journaling has been used as a self-reflective practice and research tool in a variety of areas, including students’ development of problem-solving skills [50], content knowledge [51], lifelong learning skills [52], changes in students’ epistemological beliefs [53], project-based learning [54–56], training of teaching assistants [57], and student empowerment [30]. In Ref. [52] a guided reflection form was used for physics students to process their learning, allowing them to express their aspirations and identify goals for their physics class. The study also highlighted intersectional experiences students can bring into their reflections, such as insights to their lived experiences and identity development in physics spaces.

Other scholars in STEM education have used writing as a strategy for ameliorating outcomes rooted in marginalization via values affirmations [58–60], utility value interventions [61], and purpose-for-learning interventions [62]. For instance, one values affirmation intervention called for targeted social-psychological interventions for minoritized groups given that they experience more negative stereotypes and are at higher risk of psychological threats [58]. Using in-class writing assignments significantly increased African American students’ grades and reduced the racial achievement gaps between their White counterparts. A similar study in physics was able to show similar gains for women [59] but a replication experiment failed to come to similar conclusions [60]. When students are given the opportunity to reflect and connect the utility of the course content to their personal and community values, it affirms their sense of belonging and positively influences their intrinsic motivation and persistence in STEM [1,61,62]. Furthermore, writing about life events has been shown to have a positive impact on physical and mental health [63]. It is important to note that values affirmation and utility-values interventions take an approach that only attends to access and achievement in an existing dominant culture. In the framing of Gutierrez [39] they help students “play the game” (the dominant axis of greater access leading to greater achievement) but do not “change the game” (the critical axis of identity affirmation leading to student empowerment). While both dominant and critical elements are necessary to understand and make progress on equity issues, in this work we explore a critical approach.

### E. Conception of the Alma reflective journaling project

We are interested in using reflective journaling to center marginalized students’ voices and lived experiences as an important part of their STEM journey. When we started the Alma Project [64], student researchers realized something was missing in their introductory STEM classes. K.T. felt as he was entering a large STEM classroom that he was “zipping up a new identity,” particularly one that reflects the White and male experiences in STEM, to navigate and survive the cultural practices of learning and doing science. Reflecting on his intersectional experiences and identities, this cultural divide between *becoming* a scientist and *sustaining* his authentic self left him wondering what it meant to exist authentically in STEM spaces, a common experience of marginalized students [65]. These experiences were also reflective of Imani Davis, a Black undergraduate woman in biology and race and resistance studies, and Alegra Eroy-Reveles, a multicultural Latina faculty in chemistry. Together, they conceptualized the Alma Project, a practice-based project that uses reflective journaling in ways that connect students’ lived experiences with their purpose in pursuing a STEM degree. In Spanish, the word “alma” translates to the heart and soul. We thought the name was fitting for the project given that students were pouring their heart and soul into the narratives they were writing, as well as the stories they shared within the classroom. Moreover, the conception of the Alma Project was born out of love for our community and recognizing the strengths of students’ full humanity. More details of the initial conceptualization and implementation of the project can be found in Ref. [64].

Our decision to utilize reflective journaling was intentional because it offers students opportunities to make connections between themselves and the spaces they occupy [66]. In this project, reflective journaling is used as a form of storytelling, serving as counterstories to dominant narratives produced in physics and other STEM spaces [40,43,67]. In other words, cultivating space where students can write about their lived experiences allows them to make intentional connections that intersect both their lived experiences and the experiences they gain in STEM classrooms. Therefore, reflective journaling also serves as a tool to document, recognize, and validate the experiences of diverse students in STEM classrooms by centralizing experiential knowledge [68]. In addition, centering on diverse perspectives disrupts dominant narratives in ways that pinpoint the need to fix systemic inequality rather than a need to “fix the student.” There is also a need to understand how students’ cultural assets are sustained in STEM spaces [69,70]. It is important for both instructors and students to recognize the values and cultural assets students bring to the classroom, and collectively learn how to cultivate and celebrate them within STEM spaces.

### F. Positionality

As critical scholars examining the lived experiences of students in STEM, we acknowledge the biases and privileges we hold through our lived experiences, cultural upbringing, and professional experiences. These multiperspectives influence how we approach our data analysis and we want to provide transparency to our readers. As a research team, we bring various perspectives from our intersectional identities and our subject matter, as described below. For one, our team draws insights from the fields that we study such as biology, nursing, kinesiology, astronomy, physics, computer science, Ethnic Studies, Latinx/a/os Studies, and Asian American Studies. We also draw insights from our personal experiences and the intersections that we occupy such as Black, Latinx, Asian American, queer, international, women, and places of privileged and oppressed.

More specifically, the authors of this paper draw from the wealth of their lived experiences. *K.T*. is a queer-identifying Vietnamese-American immigrant who is the first in his family to pursue higher education. He is a doctoral candidate in biology education where he studies justice-oriented equity teaching and learning in science education. *A.M.B*. completed a two year postdoctoral fellowship with SF BUILD and is a lecturer faculty in the department of Kinesiology. She is Latina and an immigrant from Guatemala who navigated higher education as a first-generation student. She has engaged in teaching, advising and research with STEM students of color. A central focus of her research is linking research to the experiences and needs of her community. At the heart of her scholarly work is creating nurturing educational spaces where all students can reach their potential and create pathways to their education and future careers. *K.C*. is a Professor of Physics and Astronomy who transitioned from cosmology science research to physics and astronomy education research 15 years ago. She is a straight, white woman who grew up in a town founded on principles of equity and social justice, with racial, socioeconomic, and religious diversity (imperfectly) built into its institutions and infrastructure. She is creeped out by super white spaces but recognizes her privilege in being able to work and live in diverse environments that support her values for much of her career. Her role on the Alma project has been to follow the lead of the junior researchers who founded the project and carried out the research, amplifying their voices, and providing support. *A.E.-R*. started the Alma project at SF State as an Assistant Chemistry Professor. She then moved “home” to UC Santa Cruz as a Chemistry Teaching Professor, which allows her three children to see their grandparents every day. As a multicultural (Chicana / Puerto Rican / Filipina) and bilingual (English / Spanish) scientist, she uses her “insider” position to connect with, support, and help underrepresented students prepare for careers in science and health. *M.A*. joined the Alma Project after having been a participant in the pilot study and earned her undergraduate degree in physiology. She is a first-generation Latina who is working as a clinical research coordinator at a large university hospital and is interested in addressing health disparities among marginalized communities. *M.H*. joined the Alma Project after completing their undergraduate degree in physiology and is currently working on cardiovascular research. They are passionate about inclusive interventions that can increase opportunity for Black, Indigenous, and People of Color scientists, like herself. *A L.M*. is a first generation Latinx woman in STEM, where she studied cell and molecular biology and chemistry. She is currently a first year medical student at the UCLA David Geffen School of Medicine. Following college, she worked as a research associate at the University of California at San Francisco and at the NASA Ames Center. *M.P*. is a Latina who joined our project while working as a project coordinator for SF Build. She earned her Masters in Public Health and her work focuses on program and policy development that aims to address the root causes of historical inequities.

### G. Research questions

This study focuses on the cultural assets that students bring to introductory algebra-based physics, grounding our analysis of students’ reflections in a CCW framework. In the following sections, we describe our research methodology and highlight the context that this study took place in. Further, we share our implementation process of the Alma Project in two settings, the algebra-based introductory physics labs and the Supplemental Instruction (SI) program. We then discuss implications of using reflective journals as an asset-based approach. The research questions that guide our study are as follows:

1. What cultural capitals do students express in their reflective journaling essays in response to the prompt “Why am I here?” in algebra-based introductory physics courses?
2. Are there any differences in the capitals expressed by students in SI classes compared with physics lab classes?

## II. METHODOLOGY

### A. Context, demographics, and setting

This study took place at one of the most diverse universities in the country (34% of students identify as Hispanic/Latino, 26% Asian, 18% White, 6% Black/African American, etc; see Table II). As a Hispanic Serving Institution (HSI) and Asian American Native American Pacific Islander Serving Institution (AANAPISI), San Francisco State University (SFSU) is committed in honoring students’ roots, developing both their intellectual and personal growth, promoting equity, and inspiring the courage to lead, create, and innovate through a commitment to social justice. As part of committing to social justice, all undergraduate students are required to complete a course that relates to American Ethnic and Racial Minorities (AERM) as a part of their education. By taking an AERM course, students learn about the views of one or more minority groups, their historical experiences and political activism. As such, these courses expose all students to a variety of perspectives that acknowledge and respect the dignity of all ethnic groups.

Student data in this study focuses on algebra-based General Physics I, which generally serves students in life science majors and some kinesiology and geoscience majors as part of their degree requirement. Approximately 300 students enroll in General Physics I each semester, in lecture sections of up to 70 and lab sections of up to 30. Students who take the lecture course must also enroll concurrently in the lab course. General Physics I labs are taught primarily by graduate teaching assistants (GTAs) as well as lecturer (nontenure track) faculty. A common curriculum is used in the labs, which are coordinated by lecturer faculty. Students enrolled in General Physics I have an optional opportunity to enroll in one-unit Supplemental Instruction (SI) courses of approximately 20 students, which are co-facilitated by pairs of undergraduate nearpeers. Demographic characteristics for SFSU overall, biology majors, and students in the SI and lab sections in this study are summarized in Table II.

In addition to the Alma Project, the Physics and Astronomy department at SFSU is implementing a number of reforms in our introductory lab courses, including offering a 3-credit pedagogy course for GTAs starting in Fall 2018 [71] and a shift from verification-style (“cook-book”) labs to guided-inquiry labs [72] starting in Fall 2019. In addition, at the beginning of every semester we hold workshops with all instructors teaching the introductory labs, so that we are on the same page as we introduce new pedagogical approaches in the courses. In taking the pedagogy course, GTAs become familiar with and apply evidence-based, student-centered science teaching strategies, reflect on their teaching practice, and help peers identify successes and challenges in their teaching. Consistent with SFSU’s focus on social justice, equity, and inclusion, our pedagogy course includes specific goals for GTAs to become familiar with and be able to implement inclusive teaching practices, including asset-based approaches.

Supplemental Instruction courses offer supplemental materials that support students’ conceptual learning in STEM major gatekeeper courses that aim to foster a collaborative learning environment and create community through hands-on and interactive small-group work. At our university, SI courses are led, facilitated, and designed by undergraduates who have previously enrolled in both STEM and SI courses. They are trained at the beginning of each semester in designing classroom communities with an emphasis on collaboration and active learning. Many SI facilitators shared that this emphasis on collaboration cultivates a sense of community and collectivism since both these SI leaders and students are able to exchange rich dialogue about conceptual learning in STEM and the successes and challenges within their corresponding lecture and lab courses [73]. Students who enroll in SI come from diverse backgrounds and those who have previously enrolled in one SI course will usually re-enroll in other SI courses.

### B. Implementation and timeline

It is important to note that the conception of this project was rooted in a community need and it was not originally intended to be a research project. As stated earlier, the student project leads noticed something was missing in their STEM educational journey. They realized that the narratives they were learning did not reflect the diverse lived experiences of their peers at their university. To center on the lived experiences of diverse students in STEM classrooms, we began implementing reflective journaling as humanzing practice for students to share their experiences.

The project was first piloted in three SI courses in Spring 2017. At the time, K.T. and Imani Davis were both SI leaders for biology and chemistry, respectively, and student researchers with A.E.-R. They also recruited two additional SI leaders from general physics to join their pilot study, in hopes that they could support the majority of STEM majors. Our pilot team met regularly to discuss journaling questions that would prompt students to recognize their lived experiences and non-STEM identities as valuable assets in STEM spaces. Together, the four SI facilitators asked students to journal for 5 min at the start of class and spent 5 min sharing afterwards for 14 weeks. Each student was provided with a composition notebook to document their stories. At the end of the semester, the SI leaders asked students for consent to read the journals and for implementation feedback. We took time to learn from our pilot implementation and discussed ways to better design the project. In Spring 2018, we expanded the reflective journaling to 13 SI sections with more than 200 students from general physics I and II, general biology I and II, general chemistry I and II, organic chemistry, and calculus. Again, students journaled every week for 14 weeks and we asked for feedback from SI leaders and students’ consent to collect and read their journals at the end of the semester.

During this time, we received valuable feedback from the SI facilitators who participated in our two-semester pilot. Many found journaling and sharing stories beneficial and expressed the importance of discussing lived experiences within their classrooms and how their assets interplay with becoming scientists. However, SI leaders also noted that the activity often went beyond 10 min and took away class time. Given that SI courses only met once a week for 75 min, allocating 10–15 min for journaling often left SI facilitators omitting material that would otherwise be important for students to learn. Several SI leaders suggested reducing the number of reflective journaling questions to respect students’ academic time. Therefore, to gain more buy-in in future expansions of the Alma Project, we selected the four questions shown in Table III for journaling approximately once per month. By Fall 2018, the entire SI program utilized reflective journaling as part of the curriculum. The Alma Project also expanded to the introductory physics and astronomy labs. A summary of the project implementation timeline is shown in Table IV. In the SI classes, we moved away from in-class journaling to writing online reflections at home and having peer discussions during class. In the labs, which are 2 h, 45 min per week, students spend 5–10 min journaling through the course learning management system (LMS) during class and then 5–10 min on discussions, for 15 min total. In both SI and lab, grading of reflections was based on participation not content. Both new and returning SI leaders received approximately 1 h of implementation training as part of their SI program pedagogy training at the beginning of each semester. All lab instructors also received training as part of the start-of-semester workshops and new GTAs received indepth training as part of the pedagogy class.

### C. Data collection

Students enrolled in General Physics I labs and those who self-selected to be in SI Physics I reflectively journaled four times throughout the semester. As part of the lab and SI program curricula, students submitted written reflections of ~150–200 words to the university’s course management system. At the end of each semester, we asked GTAs and SI leaders whether they were willing to share written reflections. Instructors who agreed then manually enrolled one of our research team members onto the course content management system. We then gathered and anonymized all students’ reflection essays responding to “Why am I here?” We chose to analyze this prompt because of the potential breadth of student responses. From the small group discussions with students, informal conversation with instructors, and discussions among the research team, it became clear that the cultural wealth of students was important to our community, which is why we decided to analyze students’ essays using that framework. In General Physics I labs, we collected 80 journal responses from 7 sections over three semesters; in SI, we collected 100 journal responses in 7 sections over four semesters (Table V). Since General Physics I lab students often concurrently enroll in SI Physics I, we identified less than 10% of overlapping in responses through common student IDs.

### D. Data analysis procedure and validity

To analyze the “why are you here?” journal reflections, we used the CCW model by Yosso and Kanagala *et al*.’s addition to the framework to guide our analysis [42,44]. While the framework provided general cultural capitals students gain throughout their higher education journey, it was important for us to document specific cultural capitals our students were fostering through their unique lived experiences at our university. Therefore we identified additional themes emergent from students’ essays through a process of iterative thematic coding [74]. We iteratively analyzed data from the pilot study in Spring 2017, data from Spring 2018 biology, chemistry and physics SI courses, data from the General Physics I SI and lab courses, and data from other introductory astronomy and physics labs to generate our code book of cultural capitals that contextualized the experiences of STEM students specifically at our university. The data presented in this paper came from General Physics I in Spring 2018–Fall 2019.

Our research team iteratively coded students’ reflections, refining our cultural capital definitions, establishing validity primarily through use of a multimember research team. Research team members were grouped into pairs; individuals coded sets of essays, then compared codes with their partners, then compared with the larger team. The full research team debriefed [75] weekly to compare coding strategies to refine and consolidate definitions of each theme, ensuring that all research team members shared a common understanding. For those responses on which the codes differed between researchers, the selections were negotiated as needed until we came to 100% agreement on the final codes to be assigned. Additionally, the first two authors reviewed the codes of all 180 journals using our developed code book to ensure consistency. After coding all essays in our sample, we tabulated frequency counts for each cultural capital theme.

For each of our two course settings, we used a Kruskal-Wallis (KW) test [76] to determine if we could aggregate data across all semesters and course sections for each cultural capital. Specifically, we had 7 sections of lab and 7 sections of SI. We wanted to make sure the frequency count distributions of cultural capitals of all 7 sections of SI (or all 7 sections of lab) were consistent before combining them. We chose the KW test because it is a nonparametric test (i.e., one that does not require the data to be normally distributed) that can be applied to three or more sample populations (in this case sections) of different sizes. A *p* value > 0.05 indicates that the frequency distributions of cultural capitals are consistent and can be combined. The *p* values for the KW test ranged from 0.07 to 0.63 for SI and 0.16 to 0.94 for lab, indicating that the multiple sections across multiple semesters in our dataset can be combined.

## III. RESULTS

For our research questions, we were interested in understanding what cultural capitals students express in algebra-based introductory physics and how the cultural capitals expressed in a required lab compared with those expressed in an optional SI class. By comparing reflective journaling in two student settings, we were able to illuminate the cultural assets students expressed within each space.

**FIG. 1.**
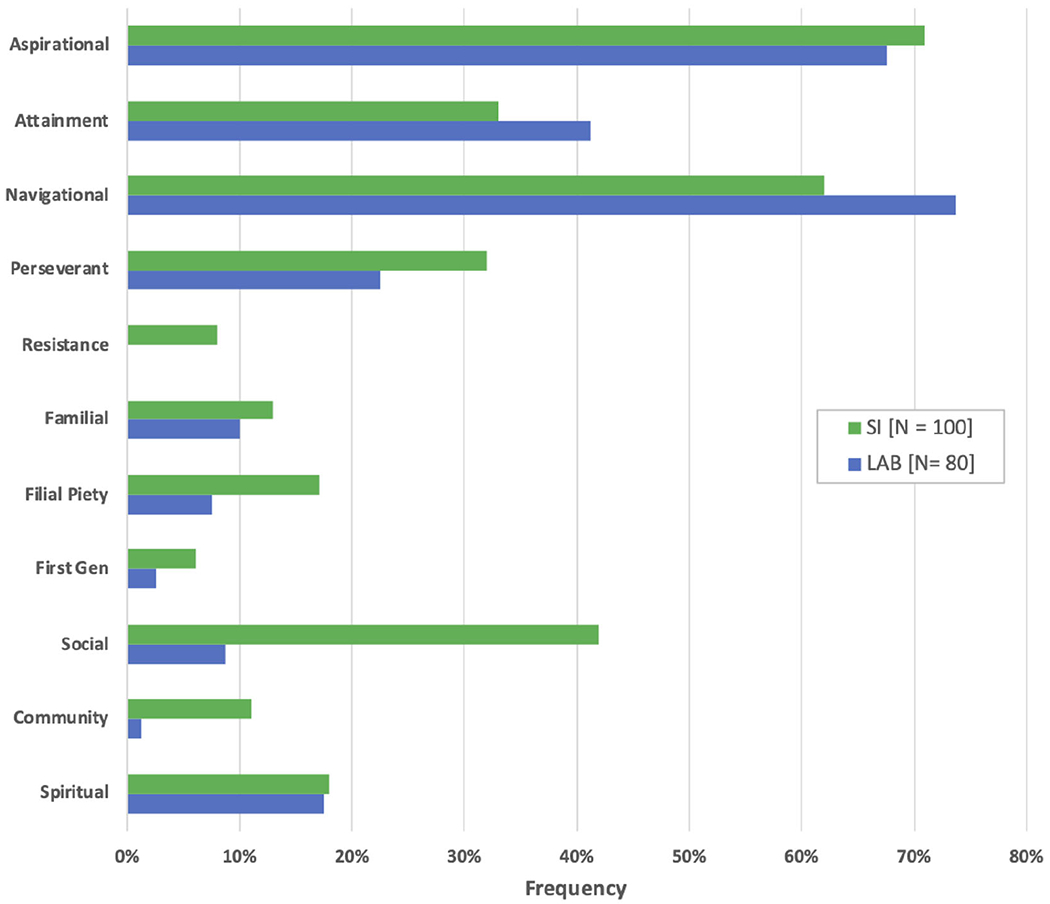
Frequency of cultural capital themes expressed in the algebra-based physics I Supplemental Instruction physics courses (green) and Introductory Physics labs (blue). There are often multiple cultural capitals expressed in a given essay, so totals exceed 100%.

Using thematic analysis, we identified 11 cultural capitals expressed by students in response to the journaling prompt “Why am I here?”. Our final coding scheme is summarized in Table VI, along with quotes illustrating each theme. We grouped our cultural capitals into four categories: (i) students’ goals: attainment and aspirational capitals, (ii) students’ experiences of the system: navigational, perseverant, resistance capitals, (iii) students’ support from and giving back to their families: familial, filial piety, first generation capitals, and (iv) students’ support from and giving back to other affinities: social, community, spiritual capitals. Figure 1 displays the frequency of each of the cultural capitals in both classroom settings.

In developing our coding scheme, we used Yosso’s conception of aspirational, navigational, familial, social, and resistant capitals and Kanagala and colleagues’ conception of perseverant and spiritual capitals. The cultural capitals that came specifically from our student population were filial piety, first-generation, community consciousness, and attainment capitals.

### A. Students’ goals: Attainment and aspirational capitals

In the goals category, aspirational capital describes the ability to maintain, cultivate, and validate personal goals, values, hopes, and dreams. Examples include a better understanding of physics, gaining broader knowledge, being able to apply knowledge, personal growth, reaching their full potential, and envisioning a better life. Attainment describes more tangible short or long-term goals, for example, obtaining a college degree or envisioning specific educational or career goals, such as becoming a physician, surgeon, physical therapist, or Ph.D.-level researcher. Aspirational and attainment capitals were among the most frequent themes and were expressed at similar rates in both student settings. Of the 80 journals from physics labs, 68% of students expressed aspirational capital; similarly, 71% of 100 journals from SI had entries relating to aspirational capital. Attainment capital was expressed by 41% of students in physics lab and 33% in SI.

### B. Students’ experiences of the system: Navigational, perseverant, resistance capitals

The category of students’ experiences of the system delves into how students navigate and deal with social institutions and higher education. Navigational capital can be defined as skills or knowledge students use to strategize paths through higher education institutions. Students frequently shared stories of navigating through college. One way of expressing navigational capital can be illustrating awareness of steps required to obtain a degree. Another example of navigating can be taking a physics course with SI as a strategy to get closer to a goal of getting a degree and recognizing that gaining specific skills are required to get into a program they desire. In this case, students are cultivating navigational capital by recognizing SI as a resource available to them that will aid in their success. Navigational capital was one of the most frequent themes, expressed by 74% of students in labs and 62% in SI. Perseverant capital describes experiences in the past or anticipation of future struggles and challenges that enable students to endure and succeed through adversity. Both communities of students highlighted struggles in STEM and are determined and self-reliant in overcoming adversities. In labs, 23% of students expressed perseverant capital, as did 32% in SI. While perseverant capital is rooted in persisting in the face of adversity, students who cultivate and express resistant capital, however, recognize systemic oppression that creates social and institutional barriers and are empowered to make change so that higher education is more accessible. In SI, 8% of students expressed resistance capital, often challenging common stereotypes about their race and gender in STEM. Resistance capital was not seen in the sample of essays from physics labs.

### C. Students’ support from and giving back to their families: Familial, filial piety, first-generation capitals

The group of capitals nurtured within students’ families often carries a sense of family history, memory, and cultural intuition. Familial capital includes support students receive from their families, whether material, emotional, or other resources, such as role modeling. In physics labs, 10% of students expressed receiving support from their families through familial capital, as did 13% of students in SI. Filial piety describes students’ sense of responsibility to their families and often serves as a source of inspiration for furthering their educational goals. It is rooted in the struggles and challenges that students know their families face, and therefore have the desire to be successful for them. Students expressed ideas of giving back to their families through filial piety in 8% of the essays from labs and in 17% of the essays from SI. While many of our students are known to be first-generation college students, we coded it as a theme in students’ essays when students specifically called it out; this occurred in 3% of the reflections from physics labs and in 6% of reflections from SI. Thus, being first generation can be seen as an asset and motivator for some students.

### D. Students’ support from and giving back to other affinities: Social, community, spiritual capitals

Parallel to receiving support from and giving back to their families, students describe receiving support from other social networks or affinities and the desire to give back to their self-defined communities and others in general. In exhibiting social capital, students recognize diverse perspectives when learning new materials as an asset and seek to cultivate networks of peers. They describe the desire to form study groups, opportunities to build new connections and be friends with others on similar paths, and cultivating relationships with instructors to learn and gain advice. They recognize the value in their peers and network in supporting their goals, furthering their learning, and making the process more enjoyable. More students in SI expressed social capital (42%), compared to students journaling in labs (9%). Community consciousness capital, in which students shared stories about their membership to a specific community in hopes of giving back, was also expressed more frequently in SI (11%) than in labs (1%). Students in SI who expressed community consciousness capital often recognized that their struggles may be similar to those within their communities and want to be part of the solution to uplift themselves and others around them. Finally, spiritual capital not only encompasses students’ faith or religion, but also gratitude, compassion, and humanitarianism as resources for hope and important motivators for students’ journeys through college and life. Students who exhibited spiritual capital described the central role of wanting to help others and make a positive difference in the world in general. Spiritual capitals were expressed at similar rates in both settings; 18% of students in labs and in SI.

### E. Overlap of cultural capitals

Students often express multiple cultural capitals in a given essay, or even in a given sentence, depicting a complex narrative. For example, attainment capital often overlapped with aspirational capital, connecting tangible end goals with an aspiration to develop oneself. As one SI physics student wrote,

“*I am here at SFSU for a better future. A future where I don’t have to struggle at the end of the month to pay bills or provide for my family* [filial piety capital]. *To achieve goals such as open up a homeless shelter* [spiritual capital] *& pay for a house for my mother so that she doesn’t have to work another day in her life* [aspirational capital]. *I aspire to become a doctor* [attainment capital] *because I know that not only can I change my life, families, but also the people around me.” (Student 12, SI physics, Spring 2018)*

In this example, the student expressed attainment capital by highlighting various aspirational goals that intersected with other forms of cultural capitals, such as filial piety and spiritual capital. Students who express attainment capital also often describe the necessary steps and actions in achieving their goals, i.e., navigational capital. As one student in lab wrote,

“*I am in physics because I need to take the class. I need to take this particular physics class because I am a biology major* [navigational capital] *and also because I was nervous about taking AP Physics in high school and decided not to do it…I had a pretty strong aversion to taking a physics class. So far, I think my nervousness was unwarranted because I feel pretty comfortable with the material that I’ve learned in class so far. I think that physics is necessary to get a really well-rounded understanding of the sciences…I am here in physics* 111/112 *at San Francisco State University because I am pursuing higher education and a science based degree [attainment capital].” (Student 16, physics lab, Fall 2018)*

In other instances, several cultural capitals would overlap within one reflection. For example, one SI physics student shared their reflection encompassing aspirational, navigational, resistance, filial piety, and community consciousness capital. They wrote,

“*I am here because I want to better myself* [aspirational capital] *& my family* [filial piety capital], *not only financially but in health. Being in this instituion [sic] will pave a way for me to become a professional* [navigational capital]. *I know that as a child I never thought of education and a career for someone who is undocumented* [resistance capital]. *I Hope to reach a position in which I can inspire and prove to all of the “immigrants” in the USA that achieving and surpassing struggle is possible* [community consciousness capital]. *Also, statistics have shown that people with higher education and wealth tend to be healthier. With my career & diploma I can improve my health & the health of my family.” (Student 3, SI physics, Spring 2018)*

Similarly, one student in lab expressed their purpose in pursuing a science degree. In their reflection, they shared experiences encompassing attainment, perseverant, filial piety, familial, and spiritual capitals. They wrote,

“*When asked the question of why I am here I think about my parents and their experience and message to me. My mom and my dad stopped going to school for a period of time before they had me and once they did have me, they took turns going back to college while working in between. During this time, they were not able to spend time with me and would have my grandparents take care of me. They were stressed out with work, school, and me. When I started high school my parents talked to me about the struggle with balancing our family along with school and work* [familial capital]. *My mom and dad cemented in me that the only way to avoid such a hectic beginning to life was to go to college right after high school, since that is what they wished they would have done* [filial piety capital]. *I see why they want me to go to school and I want to make them proud. I found my calling in the sciences and am now pursuing a career in child psychiatry* [attainment capital]. *[I] am here because I want to not only better my own life, but better the lives of others to the best of my ability* [spiritual capital].” *(Student 5, physics lab, Fall 2018)*

While these examples highlight some of the overlap between attainment capital and other cultural capitals, a full exploration of correlations between capitals and how they manifest in different classroom contexts is beyond the scope of this analysis. Exploring how cultural capitals network together can illustrate powerful narratives that highlight intersectional experiences in diverse student populations, which we aim to investigate in future studies.

## IV. DISCUSSION AND LIMITATIONS

In this study, students enrolled in General Physics I labs and SI Physics I courses engaged in reflective journaling in response to the “Why am I here” prompt. Reflective journaling, as a form of storytelling, aligns with CRT’s tenets as it centers on the lived experience of Students of Color to document their collective empowerment and leverage the knowledge and experiences they bring into each classroom [77]. As an asset-based tool, we utilized reflective journaling to center students’ voices and experiences to gain an understanding of the cultural capitals physics students expressed while navigating through STEM spaces. We used a CCW model as our theoretical framework to guide our understanding of cultural capitals and the assets students can bring into STEM spaces. We analyzed 180 reflective journal essays from both student settings and found that students expressed their cultural capitals when afforded the opportunity.

### A. Students’ assumptions and expectations of classroom environment could affect expression of cultural capitals

Students’ assumptions about the classroom environment, for instance, may affect how they express certain cultural capitals. As previously mentioned, students in both settings reflectively journal about why they were there between week 3 and 4 of the semester. Many students from both settings at this point could be influenced by their assumptions and expectations of the enrolled course to respond to the first journaling prompt. They might have utilized their assumptions and attitudes about the field (i.e., physics as a discipline or introductory physics as their first physics course) and/or feedback from their peers who have previously taken the course (i.e., a more senior student shares their feedback on SI Physics) while doing their reflections. We suspect that these prior assumptions may have influenced the way in which students share their lived experiences.

Physics students who self-selected to be in the SI program, for example, may already have a preconceived idea about SI. At the beginning of each semester, SI facilitators often visit lecture classrooms to invite students to join their course. During these course visits, SI leaders often highlight that the SI program focuses on learning STEM as a collective and active process and values community-based learning. Students then expect that SI courses will take a collective-learning approach within these spaces prior to being part of the learning community. Several students also expressed in their reflection essays that they have previously enrolled in an SI course and recognized the benefits of being part of a learning community.

On the other hand, for physics labs, students may expect to reinforce the physics concepts learned in lecture through developing practical lab skills and hopefully make meaningful connections between physics and the natural world [78]. However, the cookbook lab style that General Physics I lab utilized until Fall 2019 may have contributed to a view that dissuades students from utilizing cultural capitals such as their social network. Students may perceive the community of practice in physics labs as working collaboratively to collect experimental data to make sense of a physics phenomenon, but expect to be individually assessed through lab reports. While much of laboratory learning requires students to work with each other, students might view skill development as an individual learning process and that their social networks may not help them receive good grades. Students’ views could also be influenced by how they are assessed in the course. For example, students were only graded on participation in SI, and student reflections did not contribute to their overall grades. In physics labs, however, students were assessed on lab participation, lab reports, and participation in reflective journaling.

Thus, these initial assumptions and expectations of the classroom environment might have impacted how students express their cultural capitals at the beginning of the semester. Our results, for instance, indicated that SI students often expressed more social capital than in physics labs. While these students are a subset of students enrolled in physics labs, social capitals were more commonly expressed in SI than they were in lab spaces.

Furthermore, the SI program at our university is known to have a large enrollment of URM, first generation students, which perhaps led to some resistance and community consciousness capitals being expressed within these spaces. Moreover, since students’ expectation of SI was to be community-centered and is often occupied by URM students, students might view SI as a “safe space” for them to share certain racial, gendered, and even political experiences; hence, resistance and community consciousness capital were expressed more frequently in these spaces.

### B. Instructor buy-in, instructor support, and fidelity of implementation could affect students’ perception of reflective journaling

Buy-in from GTAs and SI facilitators could also impact how students perceive journaling in the classroom. We found it necessary to support SI and lab instructors in understanding the purpose of the Alma Project and in framing the project for students. The process of doing so played out differently in the two course settings.

**FIG. 2.**
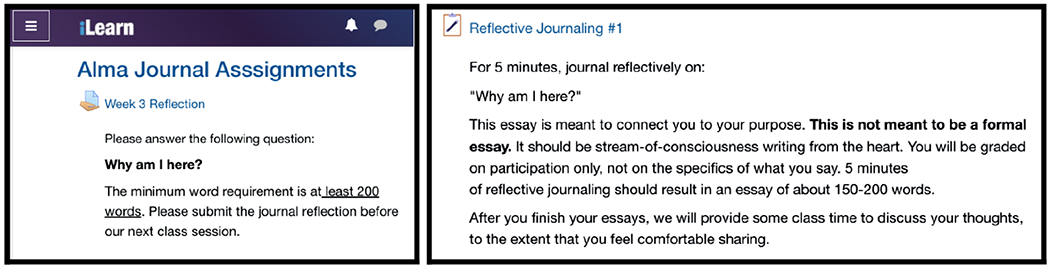
Screenshots of initial (left) and final (right) versions of instructions in the LMS.

Since the conception of the GTA pedagogy course, reflective journaling has been presented as an important way to learn what students’ motivations, goals, values, and interests are and a way for GTAs to help students feel that they belong. Implementation training took the form of modeling the process and discussions during the pedagogy course. We found areas of growth in lab instructors as a result of taking the pedagogy course, including developing a growth mindset and asset-based thinking, identifying the need for inclusive teaching practices, and implementing such practices [71]. Over several semesters we added more detailed written supports into the assignments in the course management system (Fig. 2), as short written instructions and verbal explanations from instructors were not sufficient to frame the purpose and expectations for students. We also standardized the amount of credit students would receive for participation in journaling. Finally, we added a discussion of Alma journaling in the pre-semester workshops for all lab instructors, not just in the GTA pedagogy course, as the project progressed. The low response rate seen in this initial study in the lab sections (52% vs 75% in SI) occurred before we implemented all of these supports.

On the other hand, as the Alma Project progressed into later years, many SI facilitators lost interest or did not understand the purpose. At the start, the students who led the Alma Project were participant researchers who were insiders to the SI community. During their time as SI leaders for multiple sections, they facilitated and ran the project. They provided implementation training at the start of each semester as part of SI’s pedagogy training, but more importantly, they acted as project liaisons to the other SI leaders who were implementing journaling in their course. They assisted in reminding SI leaders to include journaling in their lesson plans and supported their colleagues when asked. Unfortunately, as time progressed, our participant-researchers moved away from SI as facilitators and graduated. The SI program continued to recruit students to become SI facilitators, many of whom experienced journaling in either SI courses or other introductory STEM classes, and to provide 1 h of training during presemester workshops, but ongoing support from their colleagues was lacking. New SI leaders, therefore, may not have had sufficient and consistent enough support to understand the importance of reflective journaling in their classroom. Moreover, expanding the reflective journaling in this manner posed challenges to fidelity of implementation. Informal feedback from both SI students and SI leaders suggested greater training in understanding the purpose of reflective journaling, mindfulness, and strategies that fostered safe spaces for students to share their personal experiences would be pertinent to addressing the infidelity of implementation.

### C. Not the complete picture: Future directions

This study sought to understand what cultural capitals our students are bringing into physics classrooms. Our analysis in this study includes responses from one reflective journaling prompt at the beginning of the semester in introductory algebra-based physics for life sciences majors. This suggests a need to further understand how expressions of cultural capitals differ across physics courses for different majors and whether the ways in which capitals are expressed varies with course environment even when frequency counts are similar. Analyzing additional reflective journal responses will illuminate which students’ cultural capitals are being expressed throughout the semester. Examining how students utilize their cultural capitals throughout the semester to navigate physics would further illuminate equitable teaching strategies that center student voices. Surveys and interviews with students and instructors on their experiences with the Alma Project would illuminate the impact of asset-based teaching in STEM spaces, particularly whether reflective journaling is cultivating an inclusive space. Finally, we note that we have only examined a small fraction of our potential dataset as we are limited by the number of researchers and time required for analysis done by hand (e.g., not using a software tool). We are working with colleagues in computer science to try to automate identification of cultural capitals using natural language processing [79].

## V. CONCLUSIONS

Here, we have illustrated that reflective journaling can be used as an asset-based teaching strategy that can cultivate students’ cultural capitals while valuing their voices and validating their experiences in STEM. Reflective journaling, in this instance, afforded students the opportunity to highlight their experiential knowledge as part of their educational journey. Thus, as they described and discussed their own assets, goals, values, and interests in physics spaces, they were reminded that their rich cultural values can intersect with physics learning.

Furthermore, this study sought to understand the cultural capitals that students expressed at the beginning of the semester in their reflective journaling in General Physics I labs and SI courses. In particular, from responses to the prompt “Why am I here,” we identified 11 cultural capitals that introductory physics students often bring at the beginning of the semester. In both the required lab and optional SI settings, the most frequently expressed capitals were navigational, aspirational, and attainment capitals, followed by perseverant and spiritual capitals. We found themes related to students’ families and communities, including filial piety and community consciousness capital, which we highlight here in addition to existing CCW frameworks. We found that social capital was higher in the Supplemental Instruction setting, where community-based active learning was emphasized. This study was conducted in a diverse environment at an institution committed to social justice.

We hope instructors will recognize the wealth in students’ lived experience and leverage what students already know in our physics classrooms and other STEM spaces, in a manner that takes into account their institutional context. Reflective journaling can help students feel heard and affirmed; it is one way for instructors to humanize their students and recognize the rich experiences they bring to physics. It is essential for equitable teaching that instructors honor students’ experiences and dignity and acknowledging students’ cultural capitals can do so.

## Figures and Tables

**TABLE I.** Tenets of critical race theory.

Tenet	Description
*The intercentricity of race and racism with other forms of subordination*	This tenet posits that race and racism are part of the fabric of the United States. Students of Color experience marginalization on a micro-and macro-level, which can intersect with other forms of subordination, such as class, gender, sexuality.
*Challenges dominant ideology*	CRT examines race and power to question relationships and critically examine the status quo, where institutions operate on ideologies such as objectivity, meritocracy, color-blindness, and neoliberal agendas that only benefit those in positions of power.
*The commitment to social justice*	The commitment to racial and social justice, ultimately eradicating forms of racism and other forms of subordination. It is also a commitment to empowering groups who are marginalized as we work toward liberation from all kinds of oppression.
*The centrality of experiential knowledge*	This tenet emphasizes the importance of lived experiences in Students of Color and other subordination groups. It argues that experiential knowledge holds legitimacy, power, and is critical to understanding and analyzing racial marginalization.
*The transdisciplinary perspective*	CRT pushes beyond disciplinary bounds, drawing knowledge from multiple fields, allowing scholars to examine oppression from multiple perspectives, in both contemporary and historical contexts.

**TABLE II.** Demographic information for SFSU undergraduates overall, biology majors at SFSU in general, and the sections of General Physics I Supplemental Instruction (SI) and lab that participated in this study. Information on race or ethnicity, first-generation status, Pell eligibility, and gender illustrate the diversity of our university along several socially constructed axes as collected by our Institutional Research Office. We did not separately collect demographic information on the participants. We note the limitations of the institutional categories. SFSU is a public, masters-granting university that serves just under 30 000 students.

Race or ethnicity	SFSU overall	Biology majors	Gen. Phys I SI sections	Gen. Phys I lab sections
Latino	34%	42%	45%	40%
Asian	28%	27%	21%	29%
White	19%	16%	17%	16%
Black/African American	6%	6%	5%	5%
Two or more races	6%	5%	5%	5%
Pacific Islander/Native Hawaiian	0.5%	0.6%	2%	1%
Native American	0.2%	0.1%	0%	0%
Unknown	6%	3%	4%	3%
International	4%	1%	0%	1%
First Generation	35%	35%	39%	36%
Pell eligible	45%	45%	49%	40%
Female	56%	70%	68%	71%
Male	44%	30%	32%	29%

**TABLE III.** Final Alma Project reflective journaling prompts used in General Physics I Lab and SI courses.

Month	Prompt
1	Why am I here?
2	What do I do when life gets challenging?
3	How have the values of my community or my family (of origin or of choice) helped me navigate through college?
4	Why do I want to go into the STEM field?

**TABLE IV.** Growth of the Alma Project.

Semester	Implementation practices and revisions	SI courses participating	Physics labs participating
Spring 2017 (Pilot)	- Journal prompts each week (paper format) - Piloted in 3 Supplemental Instruction courses - <60 student participants	General Physics I General Chemistry I General Biology I	not applicable
Spring 2018	- Journal prompts each week (paper format) - 13 Supplemental Instruction sections added - >200 student participant	General Physics I, II General Biology I, II General Chemistry I, II Organic Chemistry I Calculus II	not applicable
Fall 2018+	- Journal prompts once per month collected via course management system - >400 student participants in all Supplemental Instruction sections - >1200 student participants in introductory physics and astronomy labs	General Physics I, II Calculus-based Physics I, II General Biology I, II General Chemistry I, II Organic Chemistry I Pre-Calculus Calculus I, II Introduction to Computer Programming	Conceptual Physics General Physics I, II Calculus-based Physics I, II, III Astronomy

**TABLE V.** Number of essays analyzed for the General Physics I required lab course and the optional Supplemental Instruction (SI) course.

Semester	Spring 2018	Fall 2018	Spring 2019	Fall 2019	Total
SI	22	38	18	22	100
Lab	…	19	32	29	80

**TABLE VI.** Definitions and examples of cultural capitals exhibited in reflective journal essays. These cultural capitals were derived from the original community cultural wealth model by Yosso [42], the extension of the model by Kanalaga *et al*. [44], and emergent capitals from our sample.

Category	Cultural capital	Definition	Example code
Goals	Aspirational	Desire for furthering personal goals and values, and overall hopes for the future.	*[I am here to] make myself proud & happy—getting education is a priveledge [sic], knowledge is power! I am here to change my life.*
	Attainment	Describes a tangible goal (i.e., something that could be added to a CV or resume).	*Ever since I was little I wanted to be a doctor so much that I can’t see myself doing anything else.*
Experiences with system	Navigational	Knowledge that allows the ability to navigate within distinct structures or worlds. Connect past, present, and future path.	*I find [physics] to be very interesting to me and I can see myself enjoying it for the rest of my life. However, I need to make it through a long hard path of schooling to chase this dream.*
	Perseverant	Determination, resilience, and inner confidence that help students embrace struggles and challenges and persist through them (past, present, or future).	*I find the subject challenging, so I definitely have a lot of room to grow in it. Hopefully, with more effort, I will be successful in this class.*
	Resistance	Rejection of oppressive values and stereotypes; built through experiences of micro-and macro aggressions.	*I want to show all the minorities that you can do it too. I want to break stereotypes in the workforce to help other people feel more comfortable in the field they want to go in rather than feeling they do not belong.*
Family	Familial	Support provided by family, whether material support (e.g., food, financial), emotional support, or role modeling.	*My parents came to this country to give their future children a better life than the one they had in Mexico.*
	Filial piety	Motivation to accomplish a goal based on past actions or expectations from family.	*As the oldest of 3 siblings, I want to be a role model and set a good example for my siblings.*
	First generation	Explicitly identifies as being the first in their family to attend college.	*I am here because I am a first generation student and I want to make my parents proud.*
Other affinities	Social	Describes the utilization of friends, social networks, and peer interactions to gather insight and information.	*So far this class has helped me be able to communicate with others and teach others what I know and listen to what they know. Being able to solve problems together and be able to understand the material that we are learning together helps me feel better about how I am doing.*
	Community consciousness	Solidarity with community and the desire to give back to a community one identifies as being part of.	*I am here because I care about my community and to help empower those around me*.
	Spiritual	Sense of faith, gratitude, compassion, or humanitarianism.	*Yeah, so like spiritually I feel like I’m here for a big reason, and I think it’s mainly just to help other people.*
